# Soil CO_2_ and N_2_O emissions and microbial abundances altered by temperature rise and nitrogen addition in active-layer soils of permafrost peatland

**DOI:** 10.3389/fmicb.2022.1093487

**Published:** 2022-12-13

**Authors:** Yanyu Song, Xiaofeng Cheng, Changchun Song, Mengting Li, Siqi Gao, Zhendi Liu, Jinli Gao, Xianwei Wang

**Affiliations:** ^1^Key Laboratory of Wetland Ecology and Environment, Northeast Institute of Geography and Agroecology, Chinese Academy of Sciences, Changchun, China; ^2^School of Hydraulic Engineering, Dalian University of Technology, Dalian, China; ^3^College of Tourism and Geographical Science, Jilin Normal University, Siping, China; ^4^College of Resource and Environment, University of Chinese Academy of Sciences, Beijing, China

**Keywords:** climate warming, nitrogen availability, soil microbial abundance, enzyme activity, boreal peatland

## Abstract

Changes in soil CO_2_ and N_2_O emissions due to climate change and nitrogen input will result in increased levels of atmospheric CO_2_ and N_2_O, thereby feeding back into Earth’s climate. Understanding the responses of soil carbon and nitrogen emissions mediated by microbe from permafrost peatland to temperature rising is important for modeling the regional carbon and nitrogen balance. This study conducted a laboratory incubation experiment at 15 and 20°C to observe the impact of increasing temperature on soil CO_2_ and N_2_O emissions and soil microbial abundances in permafrost peatland. An NH_4_NO_3_ solution was added to soil at a concentration of 50 mg N kg^−1^ to investigate the effect of nitrogen addition. The results indicated that elevated temperature, available nitrogen, and their combined effects significantly increased CO_2_ and N_2_O emissions in permafrost peatland. However, the temperature sensitivities of soil CO_2_ and N_2_O emissions were not affected by nitrogen addition. Warming significantly increased the abundances of methanogens, methanotrophs, and *nir*K-type denitrifiers, and the contents of soil dissolved organic carbon (DOC) and ammonia nitrogen, whereas *nir*S-type denitrifiers, β-1,4-glucosidase (βG), cellobiohydrolase (CBH), and acid phosphatase (AP) activities significantly decreased. Nitrogen addition significantly increased soil *nir*S-type denitrifiers abundances, β-1,4-N- acetylglucosaminidase (NAG) activities, and ammonia nitrogen and nitrate nitrogen contents, but significantly reduced bacterial, methanogen abundances, CBH, and AP activities. A rising temperature and nitrogen addition had synergistic effects on soil fungal and methanotroph abundances, NAG activities, and DOC and DON contents. Soil CO_2_ emissions showed a significantly positive correlation with soil fungal abundances, NAG activities, and ammonia nitrogen and nitrate nitrogen contents. Soil N_2_O emissions showed positive correlations with soil fungal, methanotroph, and *nir*K-type denitrifiers abundances, and DOC, ammonia nitrogen, and nitrate contents. These results demonstrate the importance of soil microbes, labile carbon, and nitrogen for regulating soil carbon and nitrogen emissions. The results of this study can assist simulating the effects of global climate change on carbon and nitrogen cycling in permafrost peatlands.

## Introduction

Soil carbon dioxide (CO_2_) emissions represent the second largest carbon (C) flux in terrestrial ecosystems, accounting for 70–90% of total ecosystem respiration (Schlesinger and Andrews, 2000; Cascio et al., 2017). Losses of soil C to the atmosphere through soil heterotrophic respiration play an important role in regulating atmospheric CO_2_. These losses are predicted to increase due to climate change, resulting in a positive C-climate feedback loop (Yuan et al., 2019; Dacal et al., 2022). The availability of nitrogen (N) changes the source-sink dynamics of ecosystem C by changing the soil CO_2_ flux (Cascio et al., 2017). Soils also act as an important source-sink for nitrous oxide (N_2_O; Wu et al., 2013, 2015). Climate warming and the input of N could change mineralization of soil N and N_2_O emissions (Ma et al., 2011). The increases in N_2_O emissions can cause changes in global warming potential, thus affecting the C sinks and CO_2_ emissions (Muhammad et al., 2022). However, little is known about how increases in temperature and N inputs interact to regulate soil emissions of CO_2_ and N_2_O and their temperature sensitivity. An increased comprehension of the microbial mechanisms under warming and N addition impact emissions of CO_2_ and N_2_O is vital for accurately simulating the consequences of a changing global climate on the C and N balance.

Low temperatures and nutrient concentration limited soil microbial activities and soil organic matter (SOM) decomposition (Koyama et al., 2014). An increase in temperature results in enhanced microbial growth and in the activation of the functional genes involved in C and N cycling (Xue et al., 2016; Wang et al., 2019). These result in increased soil C decomposition and respiration (Han et al., 2013). However, a previous study noted a reduction in N_2_O production with increasing temperature, especially due to denitrification (Duan et al., 2019), whereas the abundances of *amo*A, *nif*H, and *nir*K increased (Jung et al., 2011; Han et al., 2013). Warming could increase N limitation of microorganisms, which, in turn, could limit the impact of increased temperature on SOM mineralization. Previous studies found that N addition increased the abundances of C decomposition and N cycling genes (Jung et al., 2011; Wang et al., 2019), leading to a stronger positive correlation between soil available N and microbial properties exposed to elevated temperature (Huang et al., 2022). Greater insight into the impacts of warming and the addition of N on soil microorganisms can assist in improving understanding of the reactions of soil C and N emissions to a global changing climate.

Soil enzymes catalyze breakdown of high molecular weight compounds, and play important functions in SOM degradation (Yao et al., 2015), measuring their activities can provide useful indicators of soil emissions of CO_2_ and N_2_O (Chen et al., 2017). Soil enzyme activities can be used to investigate microbial nutrient cycling due to their connections with active microbial biomass, including microbial responses to environmental changes, transformation rates, and the location of the most active biomass (Wang et al., 2015). Warming can result in changes in enzyme activities, leading to functional changes in soil ecosystem processes (Xu et al., 2015). An improved understanding of decomposition and mechanisms of microbial enzyme production can assist in constraining long-term responses to warming (Sihi et al., 2016). Moreover, enzyme activities were applied as indicators of the impacts of N input within many recent experiments since they reflect the metabolic needs of soil microbial communities relative to available nutrients (Ochoa-Hueso et al., 2013). Nitrogen addition significantly stimulated activities of N- and phosphorus-acquiring hydrolytic enzymes and depressed the activities of oxidative enzymes (Tu et al., 2014). Maslov and Maslova (2021) investigated the effect of increased N availability on changes in soil enzyme activities to better understand the internal mechanisms of soil C and N cycling processes. Improved comprehension of soil enzymes and their regulatory mechanisms is needed to enhance comprehension of the impacts of temperature and N availability on soil CO_2_ and N_2_O emissions.

Peatlands represent an important C pool on Earth, storing 1,055 Gt of soil C, even though they only cover 3% of the land surface of the Earth (Nichols and Peteet, 2019). In particular, permafrost peatlands experience increased storage and emissions of C, and can act as key contributors to global warming. Permafrost thaw in northern peatlands results in alterations to ground thermal conditions, moisture, and chemistry, which, in turn, regulate microbial activities responsible for generating greenhouse gases (GHGs) from decomposing organic matter (Kirkwood et al., 2021). Newly thawed permafrost in Western Canada is predicted to release 0.2 to 25% of stored C by 2,100 (Jin and Ma, 2021). An increase in annual temperature by 1°C was predicted to increase respiration by up to 60% in an experiment conducted in Arctic blanket peatland (Dorrepaal et al., 2009). Moreover, increases in N input affected N_2_O emissions in northern peatlands due to increased N availability and/or changing vegetation composition (Le et al., 2020). Nitrogen addition could mitigate the positive effect of warming on methane fluxes in a coastal bog (Gong et al., 2021). However, the synergistic environmental parameters regulating GHGs emissions in northern permafrost peatlands remain largely unknown (AminiTabrizi et al., 2020). Clarifying the synergistic effects of both climate warming and a rising nitrogen availability on permafrost emissions of CO_2_ and N_2_O can provide a reference for future studies on potential responses of C and N sequestration of high latitude peatlands to climate change.

Northeastern China contains the second largest expanse of permafrost in China, primarily known as Xing’an-Baikal permafrost. This permafrost area lies on the southeastern edges of the Eurasian cryolithozone and is thermally unstable and sensitive to external changes (Wei et al., 2011). By the 2010s, the area of Xing’an-Baikal permafrost in Northeast China had declined by 40.6% compared with that in the 1960s (Li et al., 2021). The present study aimed to understand the synergistic effects of both climate warming and rising N availability on soil emissions of CO_2_ and N_2_O and its regulation mechanism in permafrost peatlands. An incubation experiment with temperature increase of 5°C and nitrogen addition of 50 mg N kg^−1^ was conducted in the Great Xing’an mountain peatland, Northeast China. The objectives of this research were to explore the response of CO_2_ and N_2_O emissions from permafrost peatland soil to warming and nitrogen addition, and clarify their driving mechanisms, which can help improve future predictions of responses of soil C and N cycling to climate warming.

## Materials and methods

### Site description and soil sampling

The study site of the present study is a typical permafrost peatland nearby the Tuqiang Forestry Bureau, Great Xing’an Mountain (52°44′N, 122°39′E), Heilongjiang Province, China. Average yearly temperature and average yearly precipitation are −3.9°C and 452 mm, respectively. The dominant species of plants are *Vaccinium uliginosum* L., Moench, Sphagnum spp., *Ledum palustre* L., *Eriophorum vaginatum* L., and *Chamaedaphne calyculata* L. The soil type of the study area according to the United States Department of Agriculture (USDA) classification system is Glacic Histoturbels (Soil Survey Staff, 2010). A soil sample of the active layer (0–20 cm) was obtained using a hand auger soil core sampler, which was filtered through a 2-mm sieve. The total C (TC) and total N (TN) of the soil sample before incubation experiments were 408.74 and 15.34 g kg^−1^, respectively, whereas soil moisture and pH were 77.18% and 5.49, respectively.

### Laboratory incubation

Fresh soil samples (15 g according to completely dry soil) were placed in 500-ml glass flasks and preincubated at 15°C for 7 days. NH_4_NO_3_ solution (2 ml) was uniformly added to soil at a concentration of 50 mg N kg^−1^, with four replicates prepared. Deionized water (2 ml) was added to the control treatment. The flask lids were sealed with rubber septa to allow the analysis of rates of emissions of CO_2_ and N_2_O at 15 and 20°C (maximum monthly mean temperature in July of 18.4°C). These soils were incubated continuously for 18 days. Trapped air in the jars was removed for CO_2_ and N_2_O determination at intervals of 2 h, 1, 2, 3, 5, 7, 9, 12, 15, and 18 days. Headspace gas in the jars was extracted using a 50-ml syringe with a three-way valve. The concentrations of CO_2_ and N_2_O were measured utilizing a gas chromatograph (Agilent 7890B, United States). Deionized water corresponding to the reduction in weight after each collection of gas was added. Soil samples were collected to determine soil microbial abundances, enzyme activities, and labile C and N contents at the end of incubation.

### Soil microbial abundances analysis

Soil DNA was extracted from a 300-mg subsample using a FastDNA spin Kit (MPbio, Santa Ana, CA, United States) in accordance with the manufacturer’s instructions. Bacterial 16S rRNA, fungal IST, and functional genes encoding *mcr*A*, pmo*A, *nir*S, and *nir*K were quantitatively evaluated *via* qPCR using an ABI StepOne instrument (Applied Biosystems, San Francisco, CA, United States). Supplementary Table S1 lists the primers and amplification details used in the present study. The PCR mixture contained 10 ng soil DNA, 0.4 μl primers (10 μM), and 12.5-μl of SYBR Buffer (TaKaRa, Beijing, China) in a final volume of 25 μl. qPCR standard curves were created by purifying amplicon products of functional and phylogenetic markers using a cyclic purification kit (Omega Bio-Tek, United States), ligated to the pMD18-T (TaKaRa) vector, and transforming into *Escherichia coli*. A plasmid mini kit (Omega Bio-Tek, United States) was utilized to remove the plasmids, with a standard local alignment searching tool used to identify specificity of plasmids. Standard curves were produced by plasmid serial dilution (Song et al., 2021).

### Soil enzyme activities measurement

The potential activities of acid phosphatase (AP), β-1,4-glucosidase (βG), cellobiohydrolase (CBH), and NAG were measured for absorbance using a microplate spectrophotometer. Aliquots (200 μl) of slurry (1 g fresh soil sample homogenized in 125-ml 50-mM acetate buffer, pH 8) and 50-μl of substrate solution (200 μM) were placed into 96-well microplates. Every microplate had eight replicate wells per assay, as well as negative and positive controls for quench correction. The microplates were incubated in darkness at 20°C for 4 h. Excitation and emission fluorescence were identified at 365 and 450 nm, respectively using Cell Imaging Multi-Mode Reader (BioTek Cytation 5, United States).

### Soil carbon and nitrogen content measurement

Soil ammonia nitrogen (NH_4_^+^-N), nitrate (NO_3_^−^-N), and dissolved organic N (DON) were extracted through the addition of 2 M KCl at a 1:15 ratio, followed by 1 h of shaking at 150 rpm at a temperature of 20°C. DON concentrations of soil were calculated as the difference between total dissolved N and inorganic N. Soil dissolved organic C (DOC) contents were analyzed using a Multi N/C 2100 analyzer (Analytik Jena AG, Germany) after extracting fresh soil with a 2 M KCl solution. Soil TN contents were analyzed after digestion with sulfuric acid (H_2_SO_4_) and potassium sulfate (K_2_SO_4_), with cupric sulfate (CuSO_4_) used as a catalyst. The products of digestion were subsequently analyzed using an AA3 continuous flow chemical analyzer (Seal Analytical, Germany). Quantification of soil moisture was by oven drying of fresh soil at 105°C to a constant weight. The pH of soil was measured using a 1:10 soil-deionized water slurry.

### Data analyses

Statistical analyses were performed in the SPSS 24.0 package. Results are shown as the average ± standard error. A two-way analysis of variance (ANOVA) was performed to evaluate the interactions between increasing temperature and addition of N on soil emissions of CO_2_ and N_2_O, microbial abundances, enzyme activities, and contents of soil C and N. Linear regression analysis was conducted to explore relationships between the soil CO_2_ and N_2_O emissions and soil microbial abundances, enzyme activities, and soil C and N contents.

The temperature sensitivities (*Q*_10_) of soil CO_2_ and N_2_O emission rates per 10°C were calculated as follows:


$$Q_{10}={\left(\frac{K_{2}}{K_{1}}\right)}^{\frac{10}{T_{2}-T_{1}}}$$

where *T_1_* and *T_2_* is the incubation temperatures for 15 and 20°C, respectively. *K*_1_ and *K*_2_ is the CO_2_ (mg CO_2_-C kg^−1^ d^−1^) and N_2_O (μg N_2_O-N kg^−1^ d^−1^) emission rates at 15 and 20°C, respectively.

## Results

### Emissions of soil CO_2_ and N_2_O and their sensitivity to temperature

**Figure 1:**
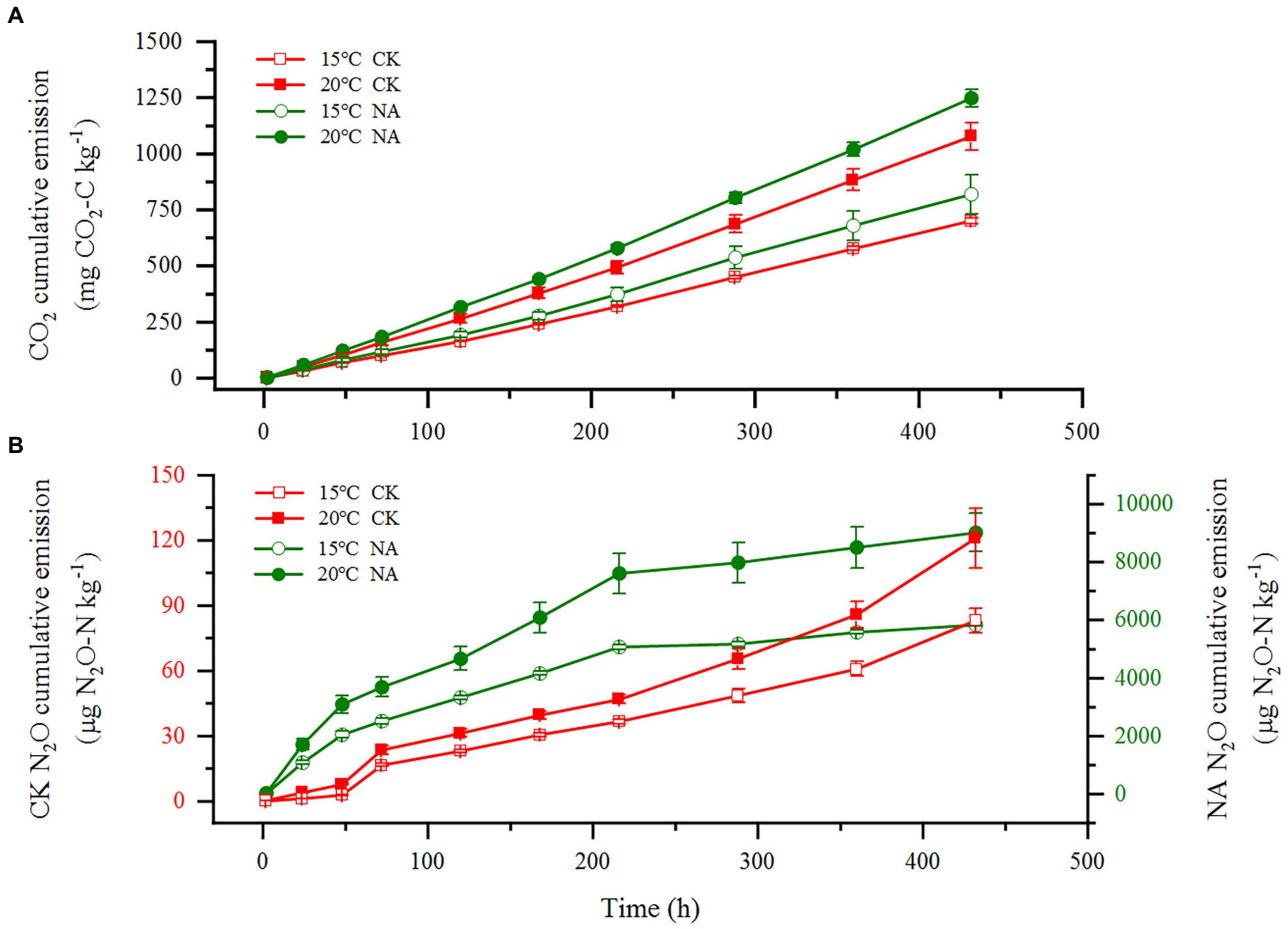
Effects of temperature rising and nitrogen addition on soil CO_2_ **(A)** and N_2_O **(B)** emissions in permafrost peatland. CK, control; NA, add 50 mg N kg^−1^ soil.

**Figure 2:**
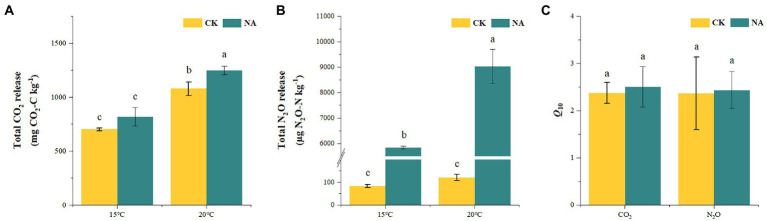
Effects of temperature rising and nitrogen addition on soil total CO_2_ **(A)** and N_2_O **(B)** release and their temperature sensitivity (*Q*_10_) **(C)** in permafrost peatland. CK, control; NA, add 50 mg N kg^−1^ soil. Different lowercase letters in the figure indicate significant differences in the means between different treatments.

An increase in temperature significantly stimulated soil emissions of CO_2_ and N_2_O in the permafrost peatlands (Figures 1A,B). Soil CO_2_ and N_2_O emissions in the control increased by 53.57 and 45.50% at 20°C compared to that at 15°C, respectively. The addition of N resulted in increases in CO_2_ and N_2_O emissions by 52.34 and 54.53% at 20°C compared to that at 15°C, respectively. The cumulative CO_2_ and N_2_O emissions were significantly higher under N addition than that in the control at 15°C and 20°C (Figures 2A,B). The increase in cumulative N_2_O emissions after the addition of N was significantly higher than the increase in CO_2_. There were significant interactions between rising temperature and addition of N on both CO_2_ and N_2_O emissions (*p* < 0.05; Table 1). The sensitivities of soil CO_2_ and N_2_O emissions to temperature in the control were 2.37 and 2.36, respectively. The addition of N did not impact the *Q*_10_ values of CO_2_ and N_2_O emissions of 2.50 and 2.44, respectively (Figure 2C).

**Table 1:** Two-way ANOVA of effects of temperature rising and nitrogen addition on soil CO_2_, N_2_O release, and soil carbon and nitrogen contents.

	CO_2_ emission rate	N_2_O emission rate	DOC	DON	NH_4_^+^-N	NO_3_^−^-N
Temperature rising	49.824**	23.030**	8.890*	0.190	65.101**	6.160*
Nitrogen addition	6.427*	476.718**	0.003	13.075**	324.349**	11.814**
Temperature rising × Nitrogen addition	0.217*	21.961**	4.935*	31.132**	2.469	1.985

DOC, dissolved organic carbon; DON, dissolved organic nitrogen. *P < 0.05; **P < 0.01.

### Soil microbial abundances

**Figure 3:**
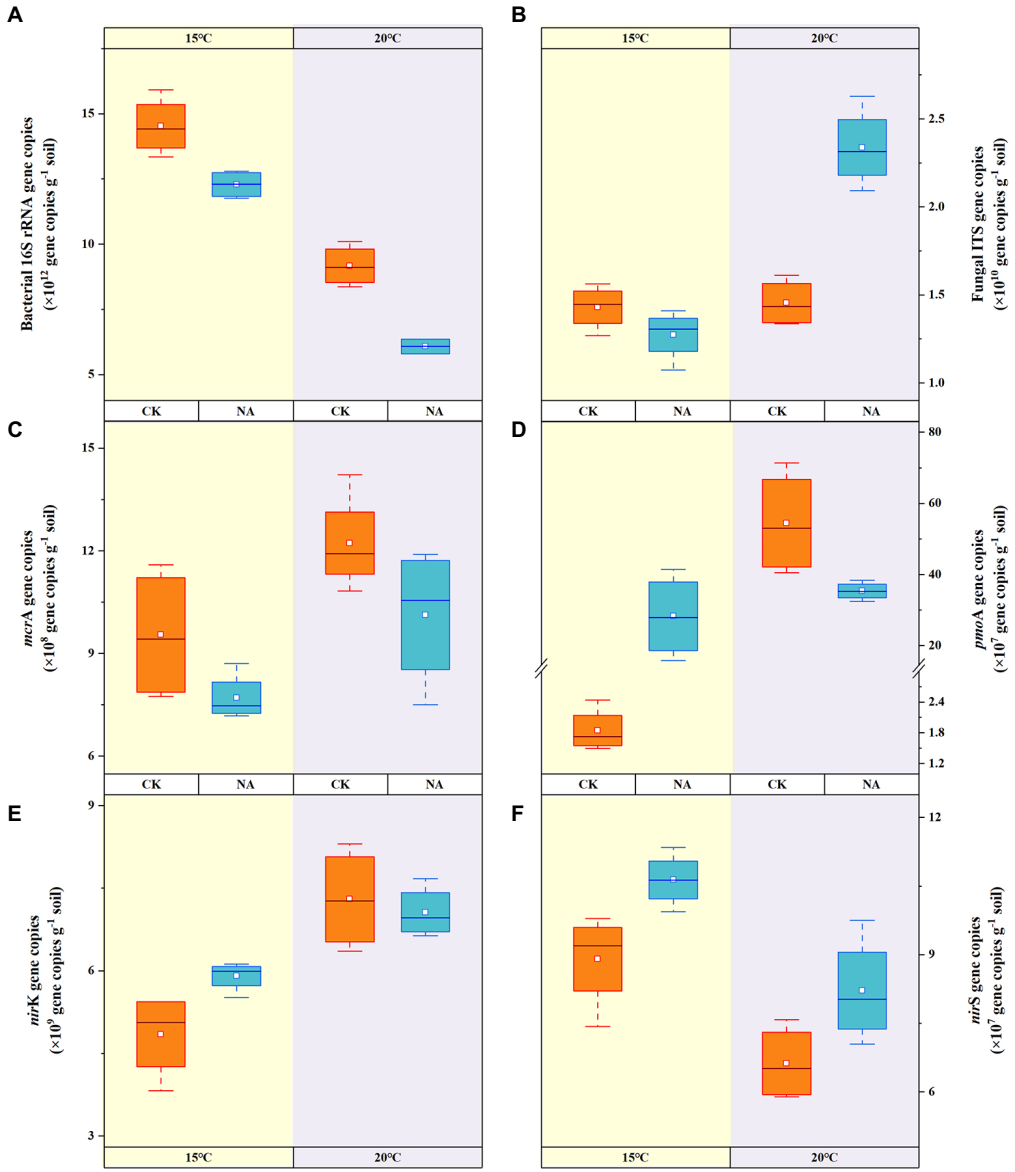
Effects of temperature rising and nitrogen addition on soil bacterial **(A)**, fungal **(B)**, *mcr*A **(C)**, *pmo*A **(D)**, *nir*K **(E)**, and *nir*S **(F)** abundances in permafrost peatland. CK, control; NA, add 50 mg N kg^−1^ soil.

**Figure 4:**
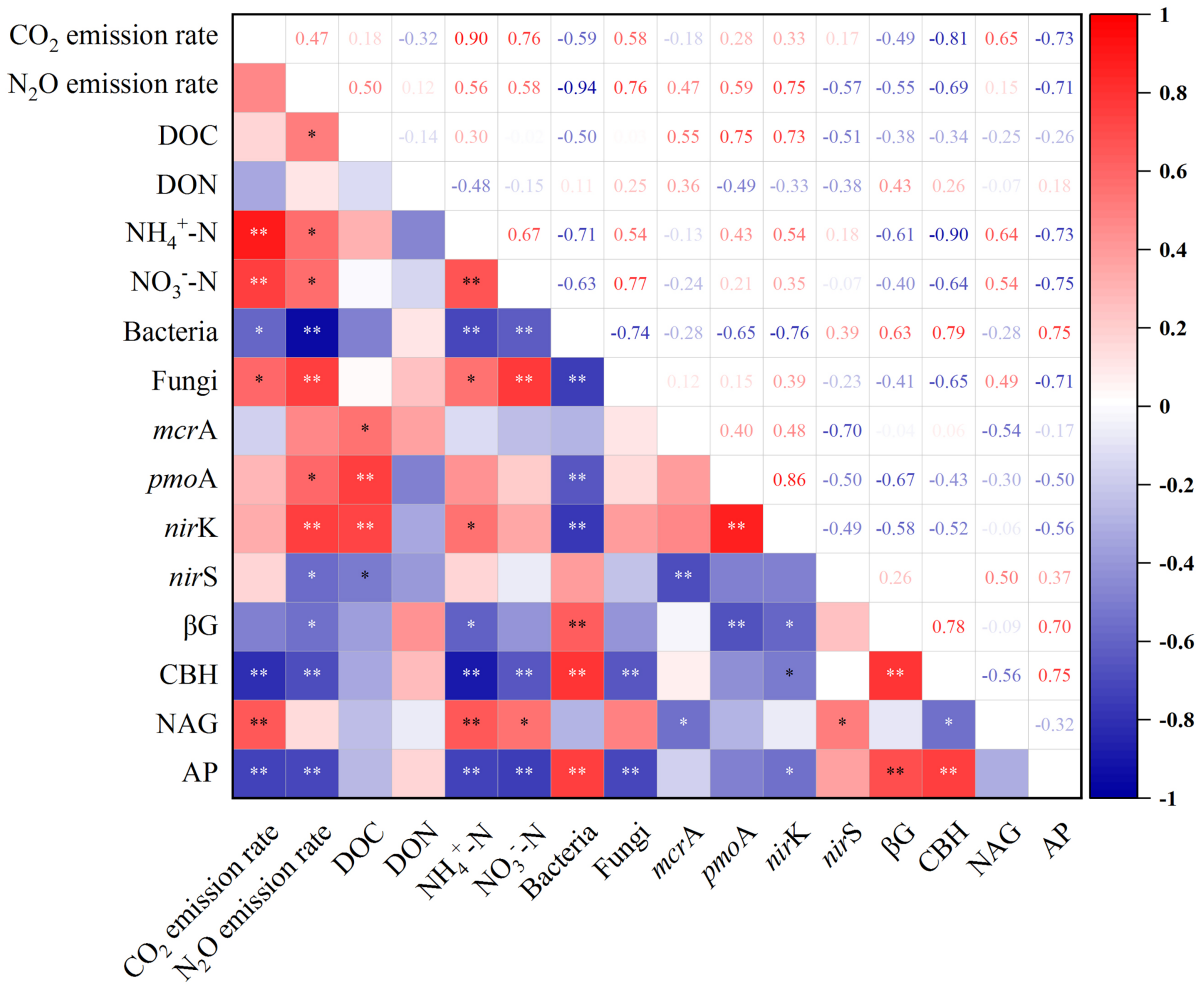
Pearson’s correlation analysis of soil CO_2_ and N_2_O emissions, carbon and nitrogen contents, microbial abundances, and enzyme activities. βG, β-1,4-glucosidase; CBH, cellobiohydrolase; NAG, β-1,4-N-acetylglucosaminidase; AP, acid phosphatase; DOC, dissolved organic carbon; DON, dissolved organic nitrogen; NH_4_^+^-N, ammonium nitrogen; NO_3_^—^N, nitrate nitrogen. * indicates significant *p* < 0.05; ** indicates significant *p* < 0.01.

Among the microbial community, bacteria were the most abundant (6.08–14.52 × 10^12^ copies g^−1^ dry soil). At 20°C, bacterial abundances in the control and N addition treatment decreased to 36.89 and 50.54% of that at 15°C (Figure 3A), respectively, indicating the preference of bacteria for lower temperature. At 20°C, fungal abundances increased significantly by 60.73% in the N addition treatment (Figure 3B). N addition appeared to reduce the abundances of bacteria under both temperatures, whereas fungal abundances were significantly stimulated at 20°C. Increased temperature resulted in the proliferation of methanogen (*mcr*A) by 28.04 and 31.46% in the control and N addition treatments, respectively (Figure 3C). However, N addition reduced methanogen abundances by 19.30 and 17.14% at 15 and 20°C, respectively. The abundances of methanotrophs (*pmo*A) significantly increased by 28.49-, 14.31-, and 18.16-fold under a rising temperature, N addition, and both increased temperature and N addition, respectively (Figure 3D). Adding N at 15°C significantly increased the abundances of *nir*K-type denitrifiers by 21.89% (Figure 3E). An increase in temperature resulted in decreases in the abundances of the *nir*S-type denitrifiers by 25.59 and 22.75% in the control and N addition treatments, respectively (Figure 3F). The addition of N resulted in increases in the abundances of *nir*S-type denitrifiers by 19.48 and 24.04% at 15 and 20°C, respectively. The increase in temperature and N addition had an interactive impact on the abundances of fungi and methanotrophs; however, there was no synergistic effect on bacterial, methanogen, and denitrifier abundances (*p* < 0.01; Table 2). There were significant relationships between the abundances of fungi and the contents of NH_4_^+^-N, NO_3_^-_^N, as well as emissions of CO_2_. This result indicated that fungi contributed to CO_2_ emissions and were affected by N concentrations. The significant correlations between N_2_O emissions and the abundances of fungi, methanotrophs, and *nir*K-type denitrifiers indicated the significant contribution of the microbial community to N_2_O emissions (*p* < 0.05; Figure 4).

**Table 2:** Two-way ANOVA of the effects of nitrogen addition and temperature rising on soil microbial abundances and enzyme activities.

	Bacteria	Fungi	*mcr*A	*pmo*A	*nir*K	*nir*S	βG	CBH	NAG	AP
Temperature rising	241.018**	45.530**	9.914**	39.356**	30.183**	25.749**	8.811*	23.166**	1.936	20.320**
Nitrogen addition	51.150**	20.357**	5.907*	0.597	1.564	12.909**	3.625	42.317**	67.208**	13.070**
Temperature rising × Nitrogen addition	1.308	41.716**	0.024	22.850**	3.897	0.024	1.226	0.630	19.659**	0.027

βG, β-1,4-glucosidase; CBH, cellobiohydrolase; NAG, β-1,4-N-acetylglucosaminidase; AP, acid phosphatase. *P < 0.05; **P < 0.01.

### Soil enzymes activities

**Figure 5:**
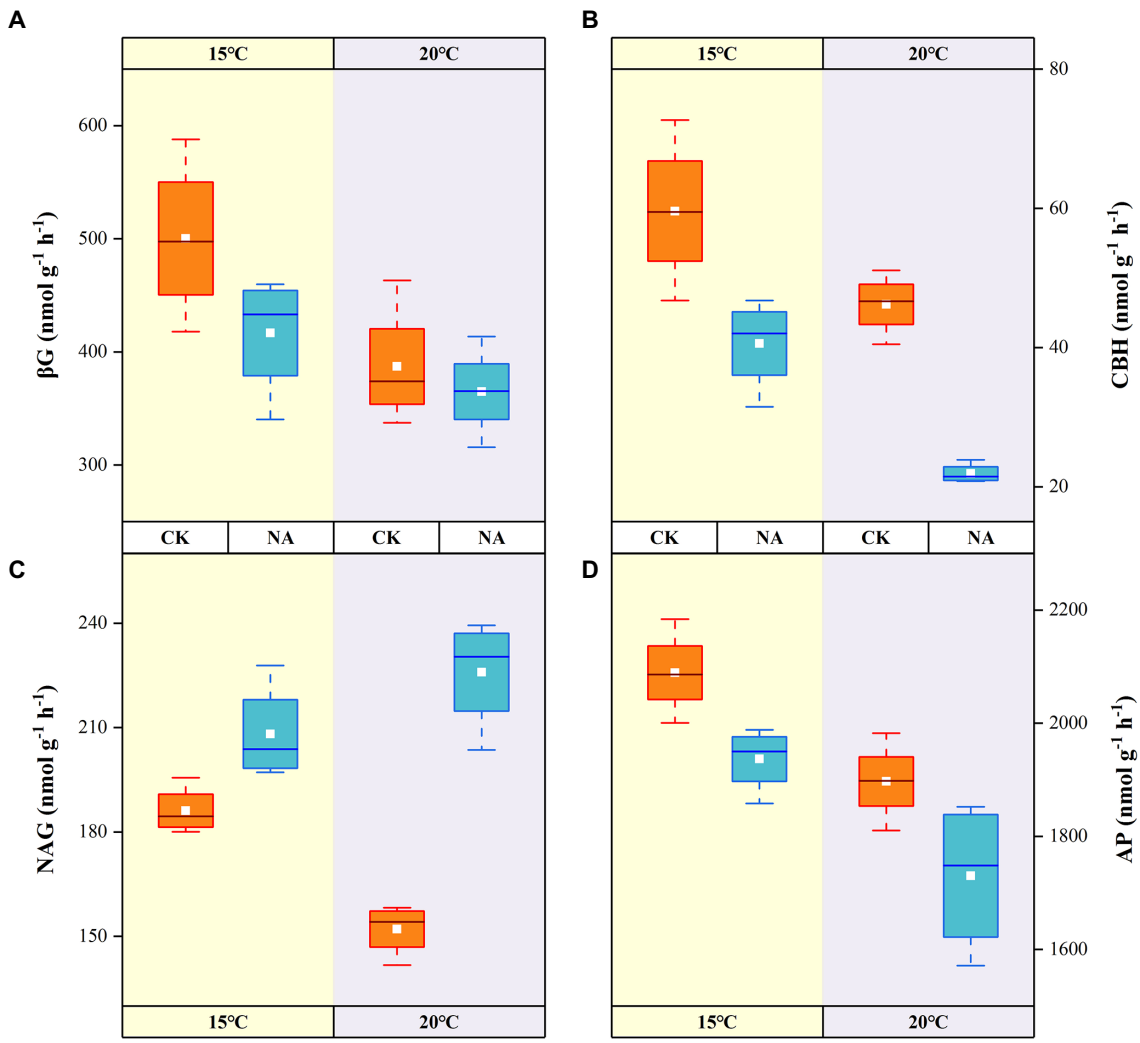
Effects of temperature rising and nitrogen addition on soil enzyme activities in permafrost peatland. CK, control; NA, add 50 mg N kg^−1^ soil. βG, β-1,4-glucosidase **(A)**; CBH, cellobiohydrolase **(B)**; NAG, β-1,4-N-acetylglucosaminidase **(C)**; AP, acid phosphatase **(D)**.

The activities of the four soil enzymes responded significantly to a rising temperature and the addition of N (Figure 5). The C-cycling-related activities of βG and CBH decreased by 22.63 and 22.46% with a rising in temperature in the control, whereas they decreased by 12.40 and 46.03% in the N addition treatment, respectively (Figures 5A,B). The rise in temperature resulted in an increase in soil NAG activities by 11.83 and 48.57% in the control and N addition treatments, respectively (Figure 5C). Significant interactive effects were observed between the rising temperature and addition of N on soil NAG activities (*p* < 0.01; Table 2). NAG activities showed significant positive correlations with soil emissions of CO_2_ and contents of NO_3_^−^-N and NH_4_^+^-N (*p* < 0.05; Figure 4). Soil AP activities decreased with a rising temperature and the addition of N, with the highest and lowest activities of 2,089.23 and 1,730.22 nmol g^−1^ h^−1^ obtained at 15°C without N addition and 20°C with N addition, respectively (Figure 5D). There were no synergistic effects of the rising temperature and addition of N on soil βG, CBH, and AP activities (*p* > 0.05; Table 2).

### Soil labile carbon and nitrogen contents

**Figure 6:**
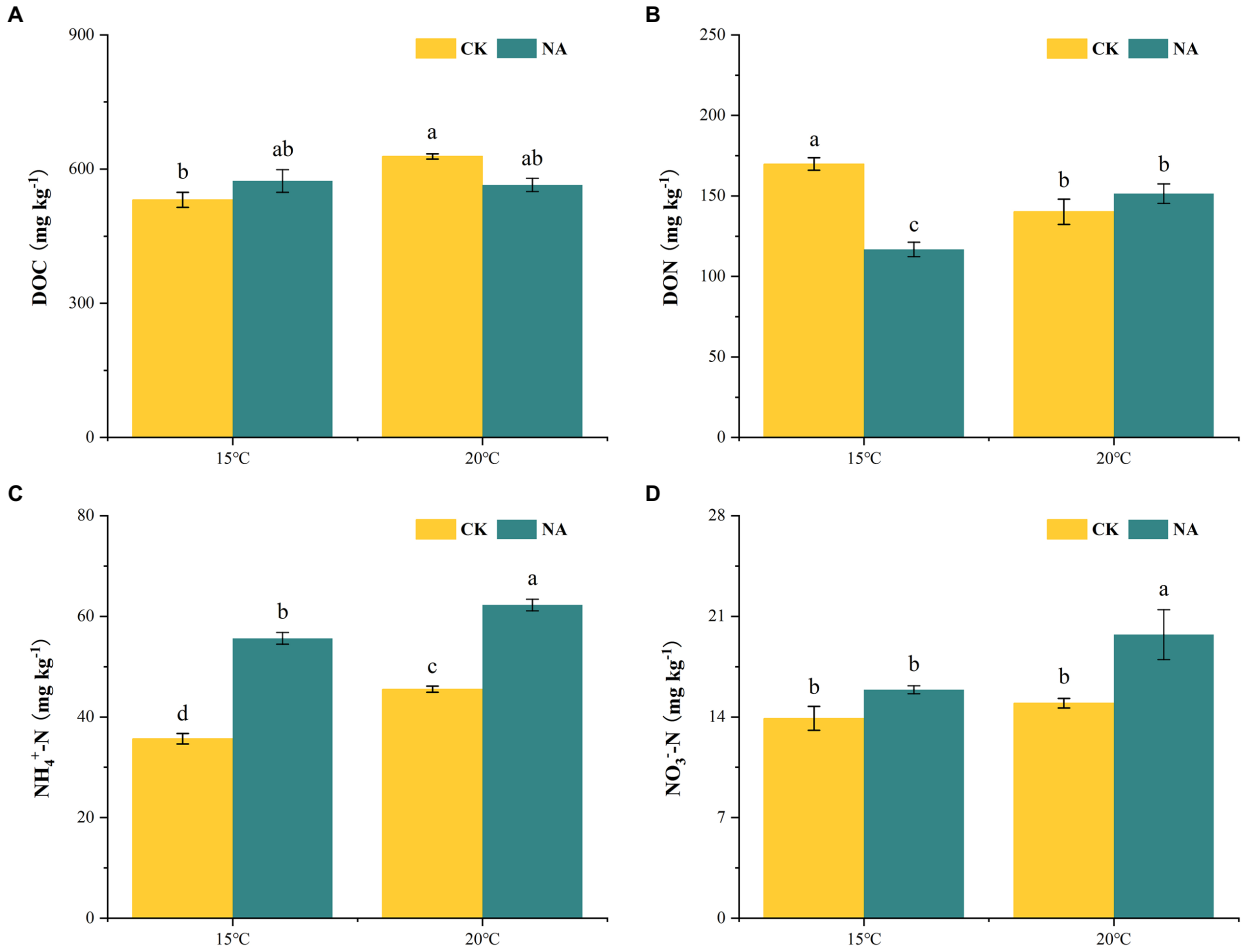
Effects of temperature rising and nitrogen addition on soil dissolved organic carbon **(A)**, dissolved organic nitrogen **(B)**, NH_4_^+^-N **(C)**, and NO_3_^−^-N **(D)** contents in peatland. CK, control; NA, add 50 mg N kg^−1^ soil; DOC, dissolved organic carbon; DON, dissolved organic nitrogen; NH_4_^+^-N, ammonium nitrogen; NO_3_^—^N, nitrate nitrogen. Different lowercase letters in the figure indicate significant differences in the means between different treatments.

An increase in temperature increased DOC contents in the permafrost peatlands from 531.05 to 628.25 mg kg^−1^ in the control treatment (Figure 6A). However, the increase in temperature did not result in a significant change in soil DOC contents under the N addition treatment. N addition had a significantly negative impact on DON contents at 15°C, with DON decreasing from 169.80 to 116.80 mg kg^−1^, whereas soil DON was not significantly affected at 20°C (Figure 6B). NH_4_^+^-N in soil ranged from 35.70 to 62.25 mg kg^−1^. Both a rise in temperature and the addition of N resulted in increased contents of soil NH_4_^+^-N (Figure 6C). The contents of soil NO_3_^−^-N under N addition (19.73 mg kg^−1^) were significantly higher than that in the control (14.96 mg kg^−1^) at 20°C (Figure 6D). The increase in temperature and N addition had significant interactive impacts on soil DOC and DON contents (*p* < 0.05; Table 1), whereas the effects on NO_3_^−^-N and NH_4_^+^-N were not significant. The soil emissions of CO_2_ and N_2_O showed significant positive correlations with contents of soil NH_4_^+^-N and NO_3_^−^-N, whereas N_2_O emissions were positively correlated with DOC contents (*p* < 0.05; Figure 4).

## Discussion

### Effect of soil microbial abundances on emissions of soil CO_2_ and N_2_O

The rise in temperature and addition of N stimulated emissions of soil CO_2_ and N_2_O. Moreover, the rise in temperature and addition of N interacted within their effect on soil emissions of CO_2_ and N_2_O. However, the results of the current study demonstrated a strong negative effect of rise in temperature and the addition of N on the abundances of bacteria. This result indicated that bacteria in permafrost peatlands were adapted to a low temperature and N-limited environment. In line with our results, warming reduced 37% of bacterial abundance and microbial metabolic capacity in the deep organic layer of an Alaska tundra (Wu et al., 2022). Our results showed that the combined effects of temperature rising and N addition significantly increased fungal abundances and there were significantly positively correlations between fungal abundances and the emissions of CO_2_ and N_2_O, suggesting that there were differences in sensitivity of different microbial communities to environmental changes and fungi communities played a vital part in the variations of CO_2_ and N_2_O emissions at higher temperature and under the addition of N. Consistent with the outcomes of the current study, Xu et al. (2017) determined that fungal tolerance to high temperatures played a significant part in N_2_O emissions.

The results of the present study showed that methanotrophs were more sensitive to a changing temperature and the addition of N compared to other microbial communities. The higher abundances of *nir*K-type denitrifiers at 20°C compared to at 15°C observed in the present study were consistent with results of previous studies in which the abundances of *nir*K genes were promoted by higher temperatures (Jung et al., 2011; Cui et al., 2016). Declines the abundances of *nir*S-type denitrifiers were observed at 20°C compared to those at 15°C. This result demonstrated that *nirS*-type denitrifiers were better adapted to low temperature conditions. The significant positive correlations between the abundances of *nir*K-type denitrifiers and NH_4_^+^-N contents and N_2_O emissions observed in the present study indicated that the increase in emissions of N_2_O could be primarily attributed to the denitrification pathway mediated by *nir*K denitrifiers. Jung et al. (2011) similarly observed an increase in *nir*K genes abundances under both warming and the addition of N. The *nir*K denitrifiers mentioned above are bacterial *nir*K, fungal *nir*K also have clear relevance for N_2_O-producing, future understanding the abundance and distribution of denitrifying fungi may provide new insight into soil N_2_O emissions under various environmental settings (Chen et al., 2016).

### Impacts of soil enzyme activities on emissions of soil CO_2_ and N_2_O

Soil enzymes play an important role in the mineralization of soil C and N. Therefore, an improved comprehension of the reaction of soil enzyme activities to increasing temperature and the availability of N is crucial for understanding the mechanisms under which emissions of soil CO_2_ and N_2_O occur. An increased temperature can alter the nutrient acquisition strategies of microbial communities. This is achieved by changing extracellular enzyme activities through the priming of decomposition of SOM, which leads to increased emissions of CO_2_ from peatlands (AminiTabrizi et al., 2022). NAG participates in N conversion and plays a significant part in the decomposition of nitrogenous substances in soil as it facilitates the degradation of chitin (Liu et al., 2019). Chitin is a major source of soil organic N. The addition of N may affect the decomposition of chitin and peptidoglycan, which, in turn, accelerates the activities of NAG (Liu et al., 2019). Consistent with the outcomes of the present study, Chen et al. (2018) and Liu et al. (2019) determined that N addition significantly increased the activities of NAG by 5.5% and 56.40–204.78%, respectively. The increase in the activities of NAG can be attributed to soil acidification induced by the addition of N. A decrease in pH was shown to positively affect soil NAG activities (Chen et al., 2018). pH is a key driver for the turnover of organic matter in cold soil, regulatory role of pH needs consideration in the future studies (Leifeld et al., 2013). Although the rise in temperature decreased NAG activities in the control treatment, the increase in NAG activities in the N addition treatment indicated that within the combined effect of an elevated temperature and addition of N, the latter had the dominant effect on soil enzyme activities.

The rise in temperature inhibited the activities of soil βG, CBH, and AP. This result could be attributed to the decrease in enzyme activities possibly being related to a decrease in substrate (e.g., microbial biomass) availability at elevated temperatures. Wang J. Y. et al. (2020) determined that enzyme activities reduced with increasing incubation time, suggesting that the responses of enzymes reflected changes in the availability of substrate due to warming. The rate of enzyme production has been shown to decrease as substrate is exhausted. The outcomes of the current study illustrated that the warming stimulation of soil respiration readily depleted hydrolysable substrates during incubation without inputs of C sources. Therefore, decreases in the active pool due to warming can result in microbial C starvation (Metcalfe, 2017; Wang J. Y. et al., 2020). In addition, bacterial conversion of NH_4_^+^-N to NO_2_^−^-N in the first step of nitrification can further acidify soils through the release of H^+^ into soil solution. Accelerated acidification, in turn, is an important factor inhibiting soil microbial enzyme activities to acute nutrient amendment (Fatemi et al., 2016). Previous studies have also suggested that a decline in soil enzyme activities was attributable to their more rapid inactivation due to warming can help explain attenuation of the warming impact on mineralization of soil C (Alvarez et al., 2018). Changes in redox conditions driven by temperature can result in abiotic destabilization of Fe-organic matter (phenol) complexes. This is a peatland decomposition pathway that was previously underestimated and can result in increased production of CO_2_ and the accumulation of polyphenol-like compounds that could further inhibit the activities of extracellular enzymes (AminiTabrizi et al., 2022).

### Effect of substrate availability on emissions of soil CO_2_ and N_2_O

The emissions of soil CO_2_ and N_2_O were related to the concentrations of NO_3_^−^-N and NH_4_^+^-N. Also, soil emissions of N_2_O were related to the concentrations of DOC. Similarly, correlations between the soil CO_2_ release and NO_3_^−^-N and NH_4_^+^-N concentrations were revealed by Zhang et al. (2018) in mountain forest and meadow ecosystems. These results indicated that higher substrate availability enhanced the activities of soil microbes, which, in turn, resulted in increased emissions of CO_2_ and N_2_O. Soil DOC is composed of low molecular weight organic compounds and drives the growth and activity of microbes by acting as an energy source and a substrate (Wang C. M. et al., 2020). The results of the present study showed an increase in DOC with increasing incubation temperature in the control. An elevated temperature accelerated microbial processes and increased C availability in the control, resulting in higher heterotrophic respiration rates and increased release of CO_2_. However, soil DOC tended to decrease with the addition of N at a higher incubation temperature, indicating that N addition may limit available C. Warming significantly increased inorganic N (NH_4_^+^-N and NO_3_^—^N; Table 1) due to higher mineralization and nitrification of TN. The above results are consistent with the earlier study of Yuan et al. (2018), and suggest that warming increases soil N mineralization. Increase in N mineralization resulted in an increase in soil available N contents with increasing incubation temperature. Higher temperatures have been shown to accelerate the denitrification and nitrification processes (Inclan et al., 2012; Zhang et al., 2016). These processes are major pathways of soil emissions or production of N_2_O (Zhang et al., 2018; Li et al., 2019). N_2_O emissions due to nitrification accounted for 60–80% of total emissions (Zhang et al., 2020). Therefore, the increased availability of C and N in the soil substrate stimulated N_2_O emissions by accelerating N transformation under warming.

In addition to soil temperature, the addition of N had profound influences on the emissions of CO_2_ and N_2_O. N addition significantly elevated NH_4_^+^-N and NO_3_^−^-N, alleviated microbial N limitation, and promoted soil CO_2_ and N_2_O emissions, thereby accelerating soil C and N cycling. Menyailoa et al. (2014) similarly found an increase in heterotrophic activity by 20–30% after the addition of N. Increase in the availability of N often accelerates soil denitrification and nitrification processes and results in increased emissions of N-oxide (Davidson et al., 2000; Benanti et al., 2014). Especially, when C are available for microbial activity, N availability will have pronounced impacts on nitrification and denitrification (Lu et al., 2015). Consistent with the result of Guo et al. (2020), the results of the present study showed a positive correlation between DOC and N_2_O emissions. This result indicated that both labile C and available N concentrations were the dominant factors influencing the emissions of N_2_O. DOC is an important factor regulating denitrification and autotrophic and heterotrophic nitrification (Ferrarini et al., 2017). DOC concentrations influence the emissions of greenhouse gasses by regulating microbial metabolism, whereas soil ammonium and nitrate do not have the same regulatory function (Chen et al., 2020). Increased C availability enhances microbial activity, and, in turn, O_2_ consumption, which may lead to sub-aerobic microsites facilitating N_2_O emissions by denitrification and nitrifier denitrification (Ma et al., 2022). Consistent with the outcomes of the current study, Zhu et al. (2016) concluded that the sensitivity of soil respiration to temperature was not influenced by the addition of N, indicating that the availability of C substrate may be more important than that of N substrate.

## Conclusion

This study showed that a rise in temperature and the addition of N promoted soil CO_2_ and N_2_O emissions. This result implies that future increases in temperature and availability of N will stimulate C and N cycling in the permafrost peatlands. The abundances of fungi were positively correlated with emissions of soil CO_2_ and N_2_O, suggesting that fungal communities may play a significant part in driving the exchange of C and N at the soil-atmosphere interface in permafrost peatlands. The abundances of the *nir*K-type denitrifiers were positively correlated with DOC and NH_4_^+^-N contents, and emissions of N_2_O, suggesting that the denitrification process mediated by *nir*K-type denitrifiers and available substrate may play a significant part in emissions of N_2_O. The activities of soil NAG increased with the addition of N and a rise in temperature, and were positively correlated with soil CO_2_ emissions. This result indicated that the activities of soil NAG are more important than those of other enzymes for regulating CO_2_ emissions. The results of the current study improve understanding of how temperature and N availability regulate soil emissions of greenhouse gasses in permafrost peatlands. However, a laboratory study cannot completely reflect the actual response of greenhouse gasses to global warming, and future research should focus on how plants and their interactions with soil microbes regulate greenhouse gas emissions under field conditions.

## Data availability statement

The raw data supporting the conclusions of this article will be made available by the authors, without undue reservation.

## Author contributions

YS: conceptualization, writing – review and editing, and funding acquisition. XC: methodology, data curation, and writing – review and editing. CS: supervision and funding acquisition. ML, ZL, JG, and XW: writing – review and editing. SG: methodology. All authors contributed to the article and approved the submitted version.

## Funding

This research was funded by the National Natural Science Foundation of China (Nos. 41871090, 41730643, and 42271109), Professional Association of the Alliance of International Science Organizations (No. ANSO-PA-2020-14), and the Innovation Team Project of Northeast Institute of Geography and Agroecology, Chinese Academy of Sciences (No. 2022CXTD02).

## Conflict of interest

The authors declare that the research was conducted in the absence of any commercial or financial relationships that could be construed as a potential conflict of interest.

## Publisher’s note

All claims expressed in this article are solely those of the authors and do not necessarily represent those of their affiliated organizations, or those of the publisher, the editors and the reviewers. Any product that may be evaluated in this article, or claim that may be made by its manufacturer, is not guaranteed or endorsed by the publisher.
