# Supplementary material for: Soil CO2 and N2O emissions and microbial abundances altered by temperature rise and nitrogen addition in active-layer soils of permafrost peatland
Source: Front Microbiol. 2022 Dec 13;13:1093487. doi: 10.3389/fmicb.2022.1093487 (PMC9792967; doi:10.3389/fmicb.2022.1093487)
Supplement: Supplementary file 1 [file Table_1.DOCX]

**Table S1 | PCR primers and amplification details used for the amplification of functional target**

| Target group | Primer | Reference | Sequence (5’—3’) | Amplification details |
| --- | --- | --- | --- | --- |
| Bacteria | Bacteria-338F  Bacteria-518R | Wang et al., 2014 | CCTACGGGAGGCAGCAG  ATTACCGCGGCTGCTGG | 95°C 2min, 35 cycles, 95°C 30s, 60°C 30s,72°C 30s, 80°C 15s |
| Fungi | ITSIF  5.8S | Gardes and Bruns et al.,1993 | TCCGTAGGTGAACCTGCGG  CGCTGCGTTCTTCATCG | 94°C 15min, 94°C 30s, 59.4°C 30s, 72°C 30s, 80°C 30s, 35 cycles |
| *mcr*A | mlas  mcrA-rev | Steinberg and Regan, 2009 | GGTGGTGTMGGDTTCACMCARTA  CGTTCATBGCGTTVGGRTAGT | 95 ^o^C 3min 30s, 36 cycles, 95 ^o^C 15s, 55 ^o^C 30s, 72 ^o^C 30s, 83 ^o^C 30s |
| *pmo*A | A189f  mb661r_nd | Holmes et al., 1999 | GGNGACTGGGACTTCTGG  CCGGMGCAACGTCYTTACC | 95 ^o^C 10min, 40cycles, 95 ^o^C 30s, 60 ^o^C 30s, 72 ^o^C 30s, 85 ^o^C 15s |
| *Bacterial-*  *nir*K | F1aCu  R3Cu | Hallin and Lindgren, 1999 | ATCATGGTSCTGCCGCG  GCCTCGATCAGRTTGTGGTT | 95°C 10min, 6 touch down cycles: 95°C 15s, 63°C 30s (-1 °C), 72°C 30s; 95°C 15s, 58°C 30s, 72°C 30s, 80°C 30s, 35cycles |
| *Bacterial-*  *nir*S | cd3aF  R3cd | Petersen et al., 2012 | GTSAACGTSAAGGARACSGG  GASTTCGGRTGSGTCTTGA | 95°C 10min, 94°C 1min, 57°C 1min, 72°C 1min, 83°C 30s, 40 cycles |

Gardes, M., and Bruns, T.D. (1993). Its primers with enhanced specificity for basidiomycetes-application of mycorrhizae and rusts. *Microb Ecol.* 2 (2), 113–118.

Hallin, S., and Lindgren, P.E. (1999). PCR detection of genes encoding nitrile reductase in denitrifying bacteria. Appl. Environ. Microbiol. 65(4), 1652–1657. Holmes, A.J., Roslev, P., Mcdonald, I.R., Iversen, N., Henriksen, K., and Murrell, J.C. (1999). Characterization of methanotrophic bacterial populations in soils showing atmospheric methane uptake. *Appl. Environ. Microb*. 65(8), 3312–3318.

Petersen, D.G., Blazewicz, S.J., Firestone, M., Herman, D.J., Donald, J., Turetsky, M., and Waldrop, M. (2012). Abundance of microbial genes associated with nitrogen cycling as indices of biogeochemical process rates across a vegetation gradient in Alaska. *Environ. Microbiol*. 14(4), 993–1008.

Steinberg, L.M., and Regan, J.M. (2009). *mcr*A-targeted real-time quantitative PCR method to examine methanogen communities*. Appl. Environ. Microb*. 75(13), 4435–4442.

Wang H., Yang J.P., Yang S.H., Yang Z.C., and Lv I.M. (2014). Effect of a 10 degrees C-elevated temperature under different water contents on the microbial community in a tea orchard soil. *Eur. J. Soil Biol*. 62, 113–120.
